# Consistency and inconsistency in network meta-analysis: concepts and models for multi-arm studies‡

**DOI:** 10.1002/jrsm.1044

**Published:** 2012-07-20

**Authors:** JPT Higgins, D Jackson, JK Barrett, G Lu, AE Ades, IR White

**Affiliations:** a MRC Biostatistics UnitCambridge, UK; b Centre for Reviews and Dissemination, University of York, YorkUK; c School of Social and Community Medicine, University of BristolBristol, UK

**Keywords:** network meta-analysis, multiple treatments meta-analysis, mixed treatment comparisons, inconsistency, incoherence

## Abstract

Meta-analyses that simultaneously compare multiple treatments (usually referred to as network meta-analyses or mixed treatment comparisons) are becoming increasingly common. An important component of a network meta-analysis is an assessment of the extent to which different sources of evidence are compatible, both substantively and statistically. A simple indirect comparison may be confounded if the studies involving one of the treatments of interest are fundamentally different from the studies involving the other treatment of interest. Here, we discuss methods for addressing inconsistency of evidence from comparative studies of different treatments. We define and review basic concepts of heterogeneity and inconsistency, and attempt to introduce a distinction between ‘loop inconsistency’ and ‘design inconsistency’. We then propose that the notion of design-by-treatment interaction provides a useful general framework for investigating inconsistency. In particular, using design-by-treatment interactions successfully addresses complications that arise from the presence of multi-arm trials in an evidence network. We show how the inconsistency model proposed by Lu and Ades is a restricted version of our full design-by-treatment interaction model and that there may be several distinct Lu–Ades models for any particular data set. We introduce novel graphical methods for depicting networks of evidence, clearly depicting multi-arm trials and illustrating where there is potential for inconsistency to arise. We apply various inconsistency models to data from trials of different comparisons among four smoking cessation interventions and show that models seeking to address loop inconsistency alone can run into problems. Copyright © 2012 John Wiley & Sons, Ltd.

## 1. Introduction

Systematic reviews that compare multiple treatments are clinically more useful than those making pairwise comparisons alone because, in the right circumstances, they allow competing interventions to be ranked. Network meta-analyses involve the simultaneous analysis of both direct and indirect comparisons among multiple treatments across multiple studies, usually randomized trials (Gleser and Olkin, 1994; Higgins and Whitehead, 1996; Lumley, 2002; Lu and Ades, 2004; Caldwell *et al*., 2005; Lu and Ades, 2006; Salanti *et al*., 2008; Ioannidis, 2009). An important component of a network meta-analysis is an assessment of the extent to which different sources of evidence are comparable, both substantively and statistically. A simple indirect comparison may be confounded if the studies involving one of the treatments of interest are fundamentally different from the studies involving the other treatment of interest. Statistical conflicts of this nature have been termed incoherence (Lumley, 2002; Ioannidis, 2009) or inconsistency (Lu and Ades, 2006; Salanti *et al*., 2008) in the literature and are the principal topic of this paper.

This is the first of a pair of papers addressing inconsistency in network meta-analysis, with particular reference to studies with more than two treatments. In this paper, we address some fundamental conceptual issues and statistical models that allow for inconsistency. In the second paper, we address parameter estimation and provide a detailed application (White *et al*., 2012). In the present paper, we first review the concept of inconsistency, discussing its relationship with the familiar notion of heterogeneity in meta-analysis. We attempt to distinguish between different types of inconsistency, which we call ‘loop inconsistency’ and ‘design inconsistency’. The distinction between these, however, is not clearly defined when there are multi-arm trials. In particular, loop inconsistency cannot be defined unambiguously when such studies are included in the analysis. Therefore, we propose a general model to allow for inconsistency that encompasses both of these types of inconsistency but does not distinguish between them. The principal message of the paper is that this general model provides an approach to identifying inconsistencies that is free of arbitrary assumptions about the nature of any evidence inconsistency. We illustrate the models using an example of a network involving smoking cessation treatments. Within the paper, we introduce novel graphical methods for depicting networks of evidence, clearly depicting multi-arm trials and illustrating where there is potential for inconsistency to arise.

## 2. Concepts of heterogeneity and inconsistency

### 2.1. Indirect comparisons

Consider trial 1, a two-arm trial of the comparison ‘B–A’, and trial 2, a two-arm trial of the comparison ‘C–B’. If the estimated effect sizes in these trials are 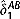 in trial 1 and 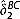 in trial 2, then an *indirect comparison* of ‘C–A’ may be obtained as 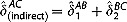. The indirect comparison maintains the benefits of randomization within each trial and allows for differences across the trials (for example, in baseline risk), provided that these differences affect only the prognosis of the participants and not their response to treatment (in whichever metric is chosen as a measure of effect size). The indirect comparison does rest, however, on the assumption that the treatment labelled as *B* is the same in both trials, so that its effects are cancelled out when ‘B–A’ and ‘C–B’ are added together. Whether the indirect comparison is truly reflective of the difference between A and C is not testable in the absence of further information. A third trial of ‘C–A’ (yielding result 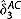) would allow us to compare the indirect comparison with a direct comparison. We say that the network of three trials is *consistent* if the underlying treatment effects are related as follows:


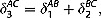


where 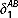, 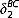 and 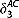 represent the true effects underlying the three studies. In practice, Equation (1) is very unlikely to hold for a particular set of three trials such as those described earlier. The reason for this may be argued either in terms of *heterogeneity* (because, within each treatment comparison, each individual study is not fully representative of all studies of that particular comparison) or in terms of *inconsistency* (because, across treatment comparisons, there are important differences in the types of studies contributing to the comparisons). We elaborate on these two concepts, among others, in subsequent sections.

### 2.2. Heterogeneity

Heterogeneity in meta-analysis is widely discussed and refers to the situation in which multiple studies of the same research question have different underlying values of the effect measure being estimated. In the network meta-analysis scenario, heterogeneity may be understood by holding the treatment comparison constant and varying the study index. In particular, heterogeneity may be said to be present for comparison ‘B–A’ if 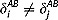 for some pair of studies *i* and *j*. Heterogeneity has been argued to be inevitable in a meta-analysis (Higgins, 2008), implying that two trials of the same pairwise comparison are unlikely to have equal underlying treatment effects. Thus, in the context of Equation (1), the equality is unlikely to hold because the particular instance of ‘C–A’ investigated in trial 3 is unlikely to represent all instances of ‘C–A’ comparisons (and similarly for trials 1 and 2 for their respective treatment comparisons).

A common way to allow for heterogeneity is through a random-effects model. This assumes that the underlying effects in multiple studies of the same comparison come from a common distribution, usually a normal distribution, that is,


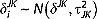


for pairwise comparison *JK* (taking values *AB*, *AC* or *BC* in the running example).

### 2.3. Consistency

The desirable relationship between direct and indirect sources of evidence for a single comparison is typically expressed in terms of a *consistency equation*


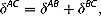


where *δ*^*JK*^ parameters represent the mean effect size across all studies of comparison *JK*. (Under a fixed-effect meta-analysis model in which it is assumed there is no heterogeneity, *δ*^*JK*^ represents a fixed (common) treatment effect for comparison *JK*.) We refer to evidence that meets the consistency equation as displaying *consistency*. We illustrate it in a network with only two-arm trials in Figure(a), as a triangle of relationships with three (nontouching) solid edges. Each edge represents one or more two-arm trials comparing the two treatments identified at either end of the edge. We draw all three edges by using the same line style (a solid line) to depict the situation in which there is no conflict (inconsistency) among them, that is, when Equation (3) holds.

**Figure 1:**
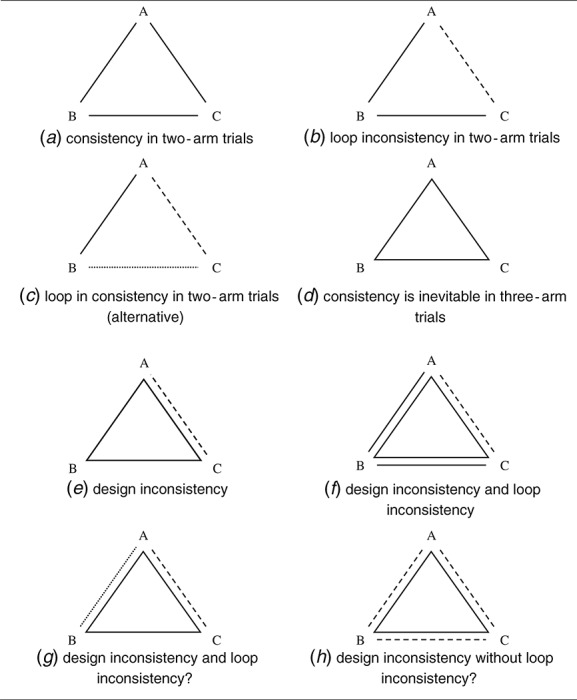
Graphical depiction of consistency, loop inconsistency and design inconsistency. (a) consistency: there is no conflict among the three sources of evidence from pairwise trials (on A vs B, A vs C and B vs C); (b) loop inconsistency: evidence on the direct comparison of A vs C (dashed line) conflicts with evidence drawn via the indirect comparison involving A vs B and B vs C (solid lines). (c) loop inconsistency: alternative scenario, indistinguishable from (b) without additional evidence; (d) consistency: three-arm trials, in which consistency is inevitable; (e) design inconsistency: evidence from the three-arm trial(s) is inconsistent with that from the two-arm trials; (f) design inconsistency and loop inconsistency: pairwise trials display loop inconsistency, whereas the three-arm trial conflicts with at least one pairwise trial, reflecting design inconsistency; and (g) design inconsistency without loop inconsistency: evidence from the three-arm trial(s) is inconsistent with that from the two-arm trials, which are themselves consistent

### 2.4. Loop inconsistency

The consistency equation (3) may not hold if studies of different treatment comparisons are substantially different in ways that affect their effect sizes, so that the effect sizes do not ‘add up’ around the loop in the figure. We call this *loop inconsistency* and depict it by drawing edges in different line styles (Figure 1(b)). Loop inconsistency can arise only from at least three separate sets of studies making different comparisons (for example, ‘B–A’, ‘C–A’ and ‘C–B’ studies). Equivalently, it can arise only when both indirect and direct estimates of an effect size are available (for example, when ‘C–B’ is measured both directly and via ‘A’ as an indirect estimate). Examples of causes of loop inconsistency are the following:

1. participants in head-to-head studies of ‘C–A’ are different from those in studies of ‘B–A’ and of ‘C–B’, for example, because they are contraindicated for treatment B, and these differences are associated with the magnitude of treatment effect;
2. versions of treatment B are different in studies of ‘B–A’ and studies of ‘C–B’ (for example, because of different doses) in ways that are associated with the magnitude of treatment effect, so that the sum of ‘B–A’ and ‘C–B’ does not equate to ‘C–A’; and
3. studies of different comparisons were undertaken in different periods, different settings or different contexts (e.g. studies of ‘C–B’ are recent, but studies involving A, a historical standard or placebo, are old), and these differences are associated with the magnitude of treatment effect.

Note that the placement of the single dashed line in Figure(b) is arbitrary from a statistical point of view, because the inconsistency is a property of the loop rather than any particular pairwise comparison. Furthermore, the three edges could be drawn in three different line styles, as in Figure(c), to indicate that different effect modifiers are associated with each edge in the loop. The distinction between the situations in Figure(b) and (c) cannot be tested statistically and would have to be informed by expert judgement.

Loop inconsistency may be regarded as a special type of heterogeneity, by imagining that every study had included every treatment. Because neither heterogeneity nor inconsistency can occur within a study, the presence of loop inconsistency as in Figure(b) or (c) implies that heterogeneity must exist in at least one of the pairwise comparisons from these imaginary ‘complete’ studies.

### 2.5. Multi-arm trials

Often, a network meta-analysis will include some studies with more than two treatment arms. Indeed, approximately a quarter of randomized trials include more than two arms (Chan and Altman, 2005), so appropriate methods to deal with the situation are important. The presence of multi-arm trials in an evidence network complicates the definition of loop inconsistency. Loop inconsistency cannot occur within a multi-arm trial. Consequently, a network may be consistent either structurally (because all studies include all treatments), or by observation (when assumptions around equality of direct and various indirect comparisons hold across studies), or through a combination of the two.

Furthermore, loop inconsistency can no longer be conveniently defined using Equation (3), because the mean effect sizes, *δ*^*JK*^, refer to pairwise comparisons from a combination of possibly inconsistent loops (e.g. from two-arm trials) as well as inherently consistent loops (i.e. from multi-arm trials). In our illustrations, we depict multi-arm trials by using a closed (joined-up) polygon (Figure(d)).

### 2.6. Design inconsistency

By the ‘design’ of a study, we mean the set of treatments compared within the study, acknowledging that this differs from conventional interpretations of the term. Design inconsistency then refers to differences in effect sizes between studies involving different sets of treatments. In allowing for this difference, we implicitly assume that different designs (i.e. different sets of included treatments) may serve as a proxy for one or more important effect modifiers. Design inconsistency is illustrated in Figure(e), where potential conflicts between study designs are again represented by different line styles. The AC effect size in the three-arm trial, drawn as a solid line, differs from the AC effect size in the two-arm trial, drawn as dashed line. Design inconsistency may also be viewed as a special case of heterogeneity, because the study designs correspond to a study-level covariate that modifies the effect sizes within the study, as in a standard meta-regression analysis. Note that, in a network consisting only of two-arm studies, the notion of design inconsistency provides no added insights compared with that of loop inconsistency.

When a multi-arm trial is involved, loop inconsistency in two-arm trials implies design inconsistency (Figure(f)). This is because the multi-arm trial must be internally consistent, so there must be a difference between the effect sizes from the multi-arm trial and at least one of those of the two-arm trials: our definition of design inconsistency. The implications of design inconsistency for loop inconsistency are less clear, however. Figure(g) illustrates design inconsistency with one 3-arm trial and two 2-arm trials. A loop can be constructed by extracting the pairwise BC comparison from the three-arm trial and comparing it with the two-arm trials. However, this ignores the presence of a consistent loop within the three-arm trial, so it is unclear whether we should describe this network as displaying loop inconsistency. Furthermore, in Figure(h), the two-arm trials are consistent amongst themselves, but their effect sizes differ from those of the multi-arm trial. Does this display design inconsistency without loop inconsistency?

### 2.7. Design-by-treatment interaction

We have seen that loop inconsistency reflects the important question of whether direct and indirect evidence agree with each other, whereas design inconsistency reflects the question, perhaps of less substantive interest, of whether the particular choice of treatments in a study is associated with different effect sizes for particular contrasts. In the presence of multi-arm trials, the distinction between the two types is difficult to draw. Nevertheless, statistical models have been motivated by attempts to tease out these particular properties of an evidence network. For example, meta-regression approaches can be used to tackle design inconsistencies, and methods we describe later by Lu and Ades (2006) address loop inconsistencies. In this paper, we argue the case for a statistical model that encompasses both types of inconsistency. This is a model that includes the full set of design-by-treatment interaction terms.

## 3. Models for inconsistency

### 3.1. Design-by-treatment interaction model

The full model allowing for design-by-treatment interaction may be written as follows. Let *J* = *A*, *B*, … index treatments; *d* = 1, … index designs; and *i* = 1, … index studies. Consider a parameter 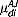 reflecting the treatment effect comparing treatment *J* with (common) reference treatment *A* in study *i*. This could represent, for example, a log odds ratio or a difference in mean responses. This framework is relevant whether arm-level data are being modelled (for example, binomial likelihoods for binary data) or whether estimates of treatment effects are being modelled (for example, log odds ratio estimates for binary data) (Salanti *et al*., 2008). To specify the model for the treatment effect parameters, it is not necessary for treatment *A* to have been included in every study. We write


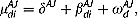


where *δ*^*AJ*^ is a fixed effect of treatment *J* relative to *A*, 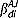 is a study-by-treatment interaction term to reflect standard heterogeneity (variability in treatment effects for comparison *AJ*, within studies of design *d*) and 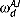 is a design-by-treatment interaction term to reflect inconsistency (variability between designs). The model in Equation (4) has the largest number of degrees of freedom (d.f.) among models allowing for inconsistency. Hence, it allows for both loop inconsistency and design inconsistency. The true effect size for comparison *AJ* in study *i* has a reference value *δ*^*AJ*^ but is subject to standard heterogeneity, represented by 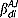, and inconsistency due to design, represented by 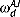. Treatment comparisons not involving *A* are defined by contrasts: 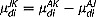. These are the *functional parameters* in the terminology of Lu and Ades (2006), whereas the 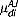 are the *basic parameters*. The functional parameters are also subject to heterogeneity and inconsistency.

We assume random effects for the standard heterogeneity, and a multivariate distribution is required across treatment effects within a multi-arm study to account for correlations between treatment effects (Salanti *et al*., 2008):


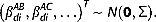


The covariance matrix, Σ, may be ‘structured’, to include assumptions about the similarity of heterogeneity variances for different pairwise comparisons; or it may be unstructured so that heterogeneity is estimated for each comparison separately; or it may lie somewhere between the two (Lu and Ades, 2009; White *et al*., 2012). A convenient structure, particularly when there is little information with which to estimate variance parameters, is to assume the same degree of heterogeneity, *τ*^2^, for every pairwise comparison in the network (Higgins and Whitehead, 1996). The between-study covariance matrix *Σ* then has diagonal elements *τ*^2^ and off-diagonal elements *τ*^2^/2.

The inconsistency parameters 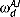 describe the perturbation in the *AJ* comparison when it takes place in studies with the specific design *d*. These may be modelled as either fixed effects (different and unrelated) or random effects (common in distribution). We prefer fixed effects, because the common distribution assumption implicit in the random-effects formulation is implausible: each 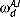 parameter has its own interpretation, and some may be *a priori* more likely to be nonzero than others. Further, the fixed-effects approach facilitates a straightforward test of the null hypothesis of consistency throughout the network of comparisons. It also allows us to interpret individual inconsistency parameters to isolate where the key conflicts in the data are occurring. From a practical point of view, using fixed effects is computationally easier and less sensitive to reparameterization than using random-effects models, and there are often too few inconsistency parameters for a random-effects model to produce reliable inferences on a random-effects variance parameter.

A disadvantage of the fixed-effects approach for inconsistency parameters is that constraints are required on the 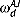 terms in order to avoid over-parameterization, and a consequence of these constraints is that the interpretation of *δ*_*AJ*_ parameters is not straightforward. In particular, the *δ*_*AJ*_ terms cannot be interpreted as treatment effects averaged over designs (as they may in the case of a random-effects assumption). The precise interpretation of the *δ*_*AJ*_ terms depends on the parameterization of the model, and care is required to ensure that the correct number of 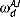 parameters is specified and that their interpretation is themselves clear.

The precise number of d.f. for inconsistency, and hence the number of nonzero 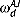 parameters, depends on the nature of multi-arm designs in the evidence network (see White *et al*., 2012, for details). An intuitive approach to specifying them is to order the designs and consider (or draw) the growing network sequentially, adding a 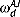 term every time a comparison is duplicated with a new design (simple design inconsistency) or when a new closed loop is formed from pairwise comparisons (loop inconsistency).

As an example, consider a network with the four designs *AB*, *AC*, *BC* and *ABC*. The full design-by-treatment interaction model is illustrated in Table 1, where the standard heterogeneity parameters and individual study subscripts, *i*, are omitted for brevity. This network has the potential for three conflicts and therefore has 3 d.f. for inconsistency, which is reflected in our use of four different line styles in the illustration. The potential conflicts may be parameterized as follows:

1. a difference in ‘B–A’ effects between *AB* studies and *ABC* studies, 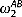;
2. a difference in ‘C–A’ effects between *AC* studies and *ABC* studies, 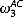; and
3. a difference in ‘C–B’ effects between *BC* studies and *ABC* studies, which could be placed on either treatment B or treatment C (we adopt a convention of placing it on the last of such possibilities, hence 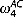 in the table).

**Table 1:** Design-by-treatment interaction model for three treatments (all possible designs). Heterogeneity terms have been omitted

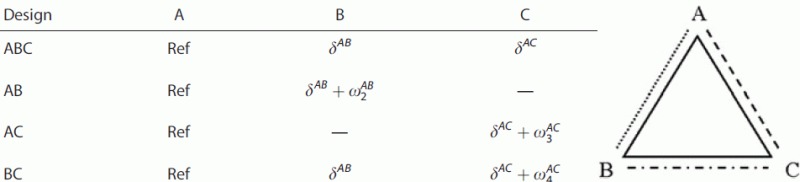

Note that this parameterization is not unique. Different parameterizations of the same model allow for different interpretations of the inconsistency parameters. For example, the following separates out a parameter to represent loop inconsistency:

1. loop inconsistency in the two-arm trials, by contrasting direct evidence ‘B–A’ in *AB* studies with indirect evidence involving ‘C–A’ from *AC* studies and ‘C–B’ from *BC* studies, 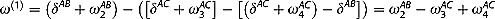;
2. design inconsistency, by contrasting ‘B–A’ effects between *AB* studies and *ABC* studies, 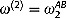; and
3. design inconsistency, by contrasting ‘C–A’ effects between *AC* studies and *ABC* studies, 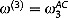.

We provide a further example of setting the 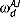 parameters later in the paper.

### 3.2. Lumley model

We note in passing that a full design-by-treatment interaction model has been proposed by Lumley, in which an inconsistency factor is introduced for each different design and all inconsistency factors are assumed to follow a common random-effects distribution (Lumley, 2002). However, the model was constructed only for two-arm trials, and extension to multi-arm trials is not immediate from the model. Lumley's model for a simple network of three pairwise comparisons is presented in Table 2. In a network of only pairwise studies, the inconsistency parameters can be viewed as being explicitly attached to specific pairwise comparisons. Our design-by-treatment interaction model would introduce only one inconsistency factor for such a data set.

**Table 2:** Lumley model for three treatments (applicable only to two-arm trials)

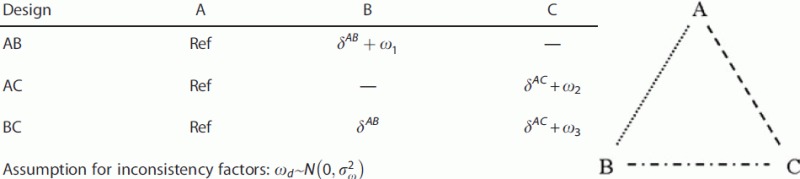

### 3.3. The Lu–Ades model

A model motivated primarily by loop inconsistency is described by Lu and Ades (2006). In this model, one inconsistency parameter is added for each independent closed loop in the evidence network (not including loops created only by multi-arm trials). Such a model for the running example is illustrated in Table 3. Lu and Ades assumed a random effect for the inconsistency parameters, whereas, in our models, we treat these as fixed effects. To specify the model, Lu and Ades first ordered the treatments (e.g. A, B and C). A general algorithm for identifying the Lu–Ades model in the presence of multi-arm studies is yet to be identified, but their examples follow the following algorithm for each design *d* and for each treatment *J* in that design:

- if design *d* includes treatment A, set 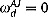 for *J* ≠ *A*;
- if design *d* includes treatment B but not A, set 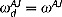 for *J* ≠ *B*;
- if design *d* includes treatment C but not A or B, set 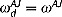 for *J* ≠ *C*; and so on.

**Table 3:** Lu and Ades model for three treatments with order *A*, *B*, *C* (all possible designs)

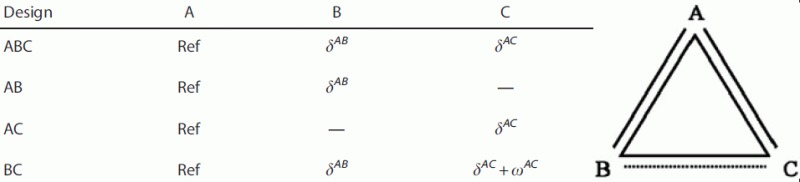

The algorithm does not guarantee that all possible independent closed loops are identified. Lu and Ades ensured that they include all closed loops by careful selection of the modelled treatment contrasts from a multi-arm trial, so that they close loops whenever possible. For example, if there are AB, AC and ABC studies in the network, then they ensured that the BC contrast is among the two modelled contrasts from an ABC study, because it forms a closed loop with the two-arm studies.

The Lu–Ades model contains a subset of the inconsistency parameters from the design-by-treatment interaction model described earlier (compare Table 3 with Table 1). The model assumptions inherent in this particular choice of treatment ordering are as follows:

- all studies containing treatment A are assumed to estimate the same treatment effects;
- all studies containing treatment B but not treatment A are assumed to estimate the same treatment effects;
- all studies containing treatment C but not treatment A or treatment B are assumed to estimate the same treatment effects; and so on.

Although basing the model on loop inconsistency is intuitively appealing, in practice, this model depends on the choice of the baseline treatment to which all other treatments are compared (treatment A in Table 3). We see from Table 3 and the previous bulleted list that an assumption is made that all studies containing the baseline treatment are estimating the same treatment effects (after allowance has been made for heterogeneity): the AB treatment differences are assumed to be the same in the two-arm and three-arm trials, as are the AC treatment differences. Thus, the model does not explicitly incorporate inconsistencies involving the first treatment (A in this example), instead it forces them to be absorbed into the heterogeneity variance. This may be a particular concern if, as is often the case, this treatment is chosen as the reference because it is the standard treatment or the one most commonly studied. The only model that contains all the Lu–Ades models (i.e. with all different treatment orderings) appears to be the design-by-treatment interaction model.

## 4. An example

To further illustrate the different models and their implications for examining inconsistency, we will apply them to example data from 24 trials investigating treatments to aid smoking cessation. These data have previously been investigated by Lu and Ades (2006) and by Hasselblad (1998). Table 4 describes the structure of the data. There are four treatments and eight study designs, two of which are three-arm designs. Note that the number of discrete lines and shapes in the figure within Table 4 is the number of designs (eight). The design-by-treatment interaction model has 1 d.f. per two-arm design and 2 d.f per three-arm design, giving 10 d.f. The consistency model has 1 d.f. per treatment, less 1, giving 3 d.f. So there are 10 – 3 = 7 d.f. for inconsistency (and hence seven 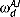 parameters). The full Lu and Ades model has 3 d.f. for inconsistency (and hence three 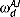 parameters), with the other 4 d.f. from the full design-by-treatment interaction model contributing instead to the heterogeneity variance.

**Table 4:** Summary of trials in the smoking data set. The graphical depiction is of a consistency model for the trials in the smoking data set, distinguishing the eight different ‘designs’ (sets of treatments included)

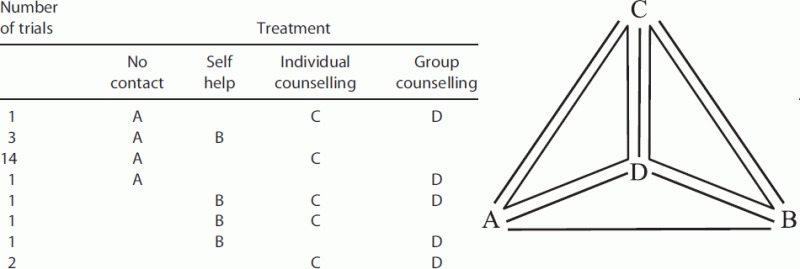

Table 5 presents a design-by-treatment interaction model for this data set. The 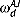 are specified using the rules outlined earlier, and we include interpretations for the inconsistency parameters, demonstrating how they can be interpreted as representing either loop inconsistency or design inconsistency. We derived Lu–Ades models by using the algorithm presented earlier. For example, for model ABCD, we have the following:

- If design *d* includes treatment A, set 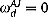 for *J* ≠ *A*; designs ACD, AB, AC and AD therefore have no inconsistency parameters.
- If design *d* includes treatment B but not A, set 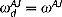 for *J* ≠ *B*; therefore, designs BCD and BC share parameter *ω*^*AC*^, and designs BCD and BD share parameter *ω*^*AD*^.
- If design *d* includes treatment C but not A or B, set 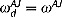 for *J* ≠ *C*; therefore, design BD has inconsistency parameter *ω*^*AD*^.

**Table 5:** A parameterization of the design-by-treatment interaction model for the smoking data set

Design	A	B	C	D	Interpretation (not unique)
ACD	Ref	—	*δ*^*AC*^	*δ*^*AD*^	
AB	Ref	*δ*^*AB*^	—	—	
AC	Ref	—	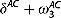	—	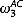 : design inconsistency for AC effect in AC compared with ACD design
AD	Ref	—	—	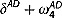	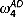 : design inconsistency for AD effect in AD compared with ACD design
BCD	Ref	*δ*^*AB*^	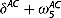	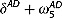	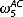 : loop inconsistency in BC (from BCD design) compared with indirect evidence from AC (from ACD design) and AB (from AB design)
					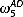 : loop inconsistency in BD (from BCD design) compared with indirect evidence from AD (from ACD design) and AB (from AB design)
BC	Ref	*δ*^*AB*^	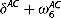	—	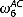 : loop inconsistency in BC (from BC design) compared with indirect evidence from AC (from ACD design) and AB (from AB design)
BD	Ref	*δ*^*AB*^	—	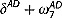	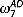 : loop inconsistency in BD (from BD design) compared with indirect evidence from AD (from ACD design) and AB (from AB design)
CD	Ref	—	*δ*^*AC*^	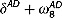	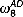 : design inconsistency for CD effect in CD compared with ACD design

Thus, the three inconsistency parameters are specified for this particular Lu–Ades model.

We fitted all models by using mvmeta, a Stata (StataCorp LP. College Station, TX, USA) macro that performs random-effects multivariate meta-regression using restricted maximum likelihood (White, 2011). The inconsistency models can be formulated as meta-regressions of *T* − 1 treatment effects (against reference treatment A) on covariates that represent the different designs. We assume a structured covariance matrix for the heterogeneity (i.e. a common variance *τ*^2^) because of the limited number of studies. Full details of different ways to implement the model are provided in our companion paper, where we also describe implementation in a Bayesian framework using the WinBUGS software. We include our Stata code in Appendix A. In our code for each Lu–Ades model, we recreate the data set by using the first treatment in the respective ordering to facilitate interpretation, although this is not strictly necessary.

Table 6 provides results from mvmeta in Stata for the design-by-treatment model and for Lu−Ades models derived from all possible treatment orderings. In fact, there are only seven unique Lu–Ades models from the 24 possible orderings of the four treatments. This is because not all designs are represented in the data set; if all possible designs had been represented, there would be 12 unique models. The ordering of the last two in the sequence is always unimportant. In Table 6, we provide chi-squared statistics and corresponding *p* values from tests for the presence of inconsistency, along with the number of inconsistency d.f. in each model and estimates of the heterogeneity variance.

**Table 6:** Tests for inconsistency for full design-by-treatment interaction model and various Lu and Ades models for smoking data set. Model ‘ABCD’ follows the algorithm as specified in Section 3.3. Other models change the order of the treatments as they appear in the algorithm. For this particular dataset, the models grouped in rows turn out to be identical models

	Inconsistency d.f.	Chi-squared	p value	Heterogeneity standard deviation (*τ*)
Full design-by-treatment interaction model	7	5.11	0.65	0.74
Lu–Ades models for different treatment orderings				
ABCD, ABDC, BACD, BADC	3	0.67	0.88	0.72
ACBD, ACDB	3	1.30	0.73	0.70
ADBC, ADCB	3	0.75	0.86	0.71
BCAD, BCDA	3	3.52	0.32	0.69
BDAC, BDCA	3	0.76	0.86	0.69
CABD, CADB, CBDA, CBAD, CDAB, CDBA	3	3.89	0.27	0.68
DABC, DACB, DBCA, DBAC, DCAB, DCBA	3	0.60	0.90	0.73

The test statistics vary considerably amongst the different Lu−Ades models. This apparently arises mainly from a design inconsistency in the ‘B–D’ effect size between the two-arm and three-arm trials. Some treatment orderings allow for this inconsistency in the model, whereas others do not. This demonstrates that the results obtained from fitting the Lu–Ades model may depend substantially on the chosen treatment ordering. In contrast, the full design-by-treatment interaction model takes account of all possible sources of inconsistency in the data. In fact, none of the models provides convincing evidence of inconsistency in this data set (*p* > 0.10 in all cases). In other examples, the particular choice of a restricted (Lu–Ades) model could have marked effects on the ability to locate inconsistencies. Interestingly, the model with the largest estimated heterogeneity variance was the one with the most d.f. (the full design-by-treatment inconsistency model). We suspect this is because, in the less complex (Lu–Ades) models, there is more information available to estimate the heterogeneity parameter, because some designs are ‘pooled’. For ‘pooled designs’ across which the design inconsistency happens to be low, this has the capacity to lower the overall estimate of the heterogeneity parameter. Such effects would not be observed if a completely unstructured heterogeneity variance–covariance matrix could have been used, but there are insufficient numbers of studies for such a model to be fitted to this data set.

## 5. Discussion

We have proposed the use of design-by-treatment interaction models as an approach to identifying inconsistencies, or conflicts, in network meta-analysis evidence structures. The approach allows for a global test for the presence of inconsistency, and models can readily be fitted in general-purpose statistical software. Our test for the presence of inconsistencies is similar to the goodness-of-fit test described by Gleser and Olkin (1994), although they did not allow for heterogeneity. We also propose more sophisticated methods for illustrating evidence networks in ways that reflect the complications introduced by multi-arm studies. Evidence inconsistency is impossible within a multi-arm study. This makes the notion of loop inconsistency awkward when there is a mixture of two-arm and multi-arm studies. We have shown how the inconsistency model proposed by Lu and Ades, which concentrates on loop inconsistencies, is a restricted version of our full design-by-treatment interaction model and that there may be several distinct Lu–Ades models for any particular data set. Our design-by-treatment approach integrates the idea of loop inconsistency with the possibility of design inconsistency, and we believe that, in the absence of clinical knowledge, it provides the only way to avoid arbitrary modelling constraints in a network meta-analysis. If there is *a priori* reason to allow for inconsistency in some places but not in others, then restricted models, including forms of the Lu–Ades model, may be appropriate.

Other approaches to assessing consistency in network meta-analyses have been proposed. Dias *et al*. (2010) described two approaches to separating direct from indirect evidence for specific treatment comparisons, motivated primarily by considerations of loop inconsistency. Lu *et al*. proposed a two-stage approach in which trials involving the same set of treatments are meta-analysed in the first stage and these results are combined at the second stage by using linear regression models. Their second-stage model is equivalent to our design-by-treatment interaction model (Lu *et al*., 2011).

The role of design-by-treatment interaction models in practice, however, requires careful evaluation. The major concern over the validity of network meta-analyses has been the possibility of loop inconsistency. Empirical studies have addressed this concern through comparison of direct with indirect sources of evidence (Gartlehner and Moore, 2008; O'Regan *et al*., 2009; Song *et al*., 2011). Little attention has been given to differences due to design (i.e. the choice of treatments included in the studies). Furthermore, pairwise meta-analyses typically ignore the presence or absence of other treatment arms in individual trials, suggesting that design inconsistency is not a major consideration in practice. The extra complexity, and accompanying loss in power in statistical tests, involved in allowing for differences across designs might therefore be unwarranted, given the low likelihood of finding important conflicts. Indeed, in our example, single studies informed estimation of several of the inconsistency factors. Although we allowed for (identical) heterogeneity to be present for all treatment effects, we might feel uncomfortable about the inferences we make with such minimal data. Conversely, the most general design-by-treatment interaction model is the only inconsistency model that does not involve arbitrary choices about where inconsistency can arise. Fitting this model, with fixed effects for inconsistency parameters, makes it, in principle, possible to locate large inconsistencies in the network wherever they are, including those that are introduced by single studies. Identification of the main sources of inconsistency may lead to well-fitting models with fewer d.f., for example, by sequential omission of apparently unnecessary inconsistency parameters.

If inconsistency of any sort is identified in an evidence network, then it remains unclear what is the best strategy to proceed. Strategies for addressing inconsistency include (i) removing portions of the evidence network; (ii) splitting nodes in the network (so that two or more different treatments replace what was previously included as a single treatment); (iii) explaining inconsistency using study-level or individual-level covariates; and (iv) seeking relevant inferences that are robust to the presence of inconsistencies (Cooper *et al*., 2009; Salanti *et al*., 2009). The degree to which useful inferences can be made from a model that includes any inconsistency terms, however, remains unclear and will be highly context specific.

## Appendix A Data and stata code for the implementation of models for smoking data (Table 6)

### A.1. Smoking data set

**Table:**

Study	Design	dA	nA	dB	nB	dC	nC	dD	nD	hasA	hasB	hasC	hasD
1	ACD	9	140	.	.	23	140	10	138	1	0	1	1
2	BCD			11	78	12	85	29	170	0	1	1	1
3	AB	79	702	77	694	.	.	.	.	1	1	0	0
4	AB	18	671	21	535	.	.	.	.	1	1	0	0
5	AB	18	116	19	146	.	.	.	.	1	1	0	0
6	AC	75	731	.	.	363	714	.	.	1	0	1	0
7	AC	2	106	.	.	9	205	.	.	1	0	1	0
8	AC	58	549	.	.	237	1561	.	.	1	0	1	0
9	AC	0	33	.	.	9	48	.	.	1	0	1	0
10	AC	3	100	.	.	31	98	.	.	1	0	1	0
11	AC	1	31	.	.	26	95	.	.	1	0	1	0
12	AC	6	39	.	.	17	77	.	.	1	0	1	0
13	AC	95	1107	.	.	134	1031	.	.	1	0	1	0
14	AC	15	187	.	.	35	504	.	.	1	0	1	0
15	AC	78	584	.	.	73	675	.	.	1	0	1	0
16	AC	69	1177	.	.	54	888	.	.	1	0	1	0
17	AC	64	642	.	.	107	761	.	.	1	0	1	0
18	AC	5	62	.	.	8	90	.	.	1	0	1	0
19	AC	20	234	.	.	34	237	.	.	1	0	1	0
20	AD	0	20	.	.	.	.	9	20	1	0	0	1
21	BC	.	.	20	49	16	43	.	.	0	1	1	0
22	BD	.	.	7	66	.	.	32	127	0	1	0	1
23	CD	.	.	.	.	12	76	20	74	0	0	1	1
24	CD	.	.	.	.	9	55	3	26	0	0	1	1
